# Views on mental health research in the context of schizophrenia: perspectives of peer support workers and researchers with lived experience – a qualitative study

**DOI:** 10.1186/s40900-026-00975-5

**Published:** 2026-09-23

**Authors:** Paul Nickel, Moritz Wigand

**Affiliations:** 1 Clinic for Psychiatry and Psychotherapy I, Centre for Psychiatry Südwürttemberg, University of Ulm, Ravensburg, Germany; 2 Clinic for Psychiatry and Psychotherapy II, Günzburg District Hospital, University of Ulm, Günzburg, Germany; 3 Faculty of Medicine, Christian-Albrechts-University of Kiel, Kiel, Germany; 4 Psychiatric and Psychotherapeutic Practice, Psychische Gesundheit am Kanal, Westerrönfeld, Germany

**Keywords:** Mental health research, Schizophrenia, Patient involvement, Patient participation, Participatory research, Qualitative research, Qualitative content analysis

## Abstract

**Background:**

Since the end of the 20th century, mental health research has been increasingly driven by the hope of uncovering the neurobiological roots of mental illness. Despite progress in this area, significant therapeutic breakthroughs — especially for complex conditions such as schizophrenia — remain limited. It is unclear whether these research priorities truly align with the needs and experiences of those affected. Greater involvement of people with lived experience in shaping research questions and processes could enhance the clinical relevance of research and lead to more effective, patient-centered interventions.

**Methods:**

This qualitative study explores the perspectives of peer support workers and researchers with lived experience in mental health research on the context of schizophrenia. We conducted semistructured interviews with 12 peer support workers and one researcher with lived experience. To contextualise and illustrate the findings, selected perspectives from 13 additional interviews with patients with schizophrenia are included. Interviews were analysed using qualitative content analysis according to Kuckartz.

**Results:**

Three central categories were identified: (1) Perception of mental health research: Participants valued both neurobiological and social approaches to research equally. They acknowledged certain advances; however, these developments were viewed as gradual and still inadequate. (2) Participation in research: Participants strongly demanded greater involvement of people with lived experience in all research stages. (3) Manner of research: Participants expressed a strong desire for user-set priorities, dissatisfaction with current research topics, perceived marginalisation, structural issues such as publication bias (e.g. favouring large randomised controlled trials over qualitative studies) and a lack of funding.

**Conclusions:**

Overall, the findings highlight the importance of incorporating lived experience in all stages of mental health research. More inclusive approaches are viewed as essential for improving research and care.

**Supplementary Information:**

The online version contains supplementary material available at https://doi.org/10.1186/s40900-026-00975-5.

## Abstract (plain language summary)

Over the past decades, mental health research has focused mainly on the biological causes of mental illness such as changes in the brain, genetics, and new technologies. Despite these efforts, real progress in treating serious mental illness such as schizophrenia has been limited. It is unclear whether this kind of research actually concerns what people with schizophrenia truly need. This study explored the views of people with lived experience of mental illness, including 12 peer support workers (people who use their own experiences to support others) and one researcher with lived experience. We conducted interviews to learn about their views on current research and what needs to be changed. Their views were enriched by interviews with 13 people with schizophrenia. The participants said that both biological and social factors are important for understanding mental health, but that progress has been slow and still does not meet people’s needs. A key finding: people with lived experience want to be involved in all parts of research – from choosing topics to sharing results. Many were also dissatisfied with current research practices. They felt that some study types are favoured over others, important topics are ignored, and there is too little funding for research that reflects real-life experiences. Overall, the study shows that including people with lived experience in mental health research could make it more useful and relevant. More inclusive approaches may help improve both the research and the care that people receive.

**Supplementary Information:**

The online version contains supplementary material available at https://doi.org/10.1186/s40900-026-00975-5.

## Background

The beginning of the 21st century in mental health research was marked by the hope of understanding the genomic causes of mental illness in order to turn the ‘decade of the brain’ of the 1990s into a ‘decade of discovery’, where new discoveries in genetics would be translated into clinical applications [1]. Research questions were and are still usually formulated by professionals [2, 3], and high-impact publications emphasise neurobiological and genetic research [4]. Despite substantial progress in these areas, significant therapeutic breakthroughs remain limited [5]. Research questions are often not driven by scientific discoveries or the needs of those affected but by social and political circumstances [6, 7]. It thus remains unclear whether research with a primarily technological or neurobiological orientation is of substantial relevance to patients themselves [2, 8, 9]. This potential misalignment between research priorities and patient needs is not unique to psychiatry. In fact, studies in somatic medicine have shown that researchers’ focus (e.g. drug trials) and patients’ interests (e.g. nondrug treatment trials) frequently mismatch [10, 11].

This misalignment is particularly problematic for chronic, severe and stigmatised illnesses. Schizophrenia is a mental illness associated with high mortality rates [12], high social follow-up costs [13], complex biopsychosocial therapy, and high readmission rates [14]. While the stigma surrounding depression is diminishing, the stigma associated with schizophrenia has remained stable (in the United States) [15] or even increased (in Germany) [16]. These challenges highlight the importance of research that aligns with patients’ needs and experiences in the context of schizophrenia.

There is growing evidence that patient involvement and taking their lived experience into account help to match research questions with research needs [9]. Within this paper, ‘Lived experience’ is understood as the subjective experience of living with a mental health condition and engaging with mental health care. Incorporating patients’ perspectives and their lived experience into scientific discourse can have several benefits: First, it may increase the frequency of implementation into practice, thus avoiding merely theoretical findings [9]. Chalmers et al. [9] suggest four recommendations to reduce unnecessary research, including user involvement in setting research priorities, thus reducing ’waste results’. There is a frequently observed lack of translation of research into practice [5, 17–20], which can also be linked to the lack of Patient and Public Involvement (PPI). A policy analysis by Wooding et al. [5] examined the translation and implementation of schizophrenia research over the past 20 years, identifying patient needs as central to achieving relevant improvements.

A growing body of literature is examining the research priorities of people with mental illness [2, 3, 21–26]. In an Australian study on research priorities, ‘the most prominent themes were experience, treatment and impact, followed by stigma, peer and trauma’ [3]. *Experience* represents a desire for a greater research focus on patients’ lived experience. *Treatment* includes topics such as recovery, care and medication, whereas *impact* describes the wish for the mental health system to have a greater impact on illness and the recovery process [3]. People with lived experience declare that medication should be complemented by social contact and peer support, as ‘medications may form part of a more complex picture’ [27]. Patients further emphasise the ‘role of social relationships, peer support and life stress’ [2].

While increasing attention is being paid to involving people with lived experience in research, less is known about their broader attitudes toward research and the research process [28, 29]. However, a scoping review by Hawke et al. revealed that the body of evidence from meta-research on PPI has increased significantly, particularly since 2024 [30]. The majority of these studies have been conducted in Anglophone countries, with none conducted in Germany [30]. The evidence from people with schizophrenia is especially limited [11, 21]. Morse et al. interviewed mental health service users, as well as carers, to gain their perspectives on ethics in mental health research [31, 32], identifying five central themes: ‘privacy and confidentiality [of the reported stories]; mechanics of conducting research; stigma; vulnerability and risk; and benefits, values, and motivations’ [31]. However, these studies focus on specific aspects of research rather than examining how research is perceived as a whole.

In summary, existing literature indicates that while research priorities are being increasingly explored, and greater PPI is indicated, there is still a lack of understanding of how people with lived experience and especially people with the diagnosis of schizophrenia perceive mental health research more broadly - particularly with regard to its focus, structure, and underlying assumptions. Addressing this gap is important for improving the relevance and impact of mental health research, shaping its future focus, and reducing ‘waste research’ [9].

This study aims to investigate the views of peer support workers and researchers with lived experience on mental health research in the context of schizophrenia. As individuals who combine personal treatment experience with professional expertise (often also in research settings) they offer a unique perspective on this subject. By exploring these perspectives, this study aims to identify potential recommendations for mental health research.

## Methods

### Design

This study is part of the project ‘Research and treatment needs in psychiatry from the perspective of people with schizophrenia, relatives and experts with experience in psychiatry’, conducted at the Department of Psychiatry Günzburg, Ulm University. Some of the findings on treatment needs have already been published elsewhere [33].

As the aim of this exploratory study is to identify new topics, questions and perspectives; a qualitative approach using content analysis according to Kuckartz [34] was chosen. Previous studies have shown that a qualitative approach is a useful way to uncover new research topics [2]. Qualitative analysis according to Kuckartz enables targeted evaluation through content analysis, while at the same time offering sufficient methodological openness to generate new topics inductively and analyse implicit meanings [34].

We used a broad concept of research needs, namely, everything that is considered necessary to be researched by the participants, especially perceived research gaps.

The study was approved by the Ethics Committee of Ulm University (approval no. 281/21).

### Recruitment

Two groups were recruited between 9/2021 and 2/2022: (1) Peer support workers and lived experience researchers were recruited as ‘experts by experience’ (EbE), a term we use in this study to describe individuals who combine lived experience with professional expertise. While all patients possess experiential knowledge, we reserve the term EbE for participants whose lived experience is explicitly integrated into a professional role. (2) Persons with a clinical diagnosis of schizophrenia or schizoaffective disorder (ICD-10: F20, F25) (hereinafter referred to collectively as patients). Findings suggest that the perspectives of different stakeholders differ with regard to key aspects of treatment [8]. EbE were chosen in the hope that their specific expertise would enable them to discuss research at a more abstract level.

Patients were continuously recruited on the open psychiatric wards of a large psychiatric hospital in southern Germany. The selection was based on the diagnosis reported by the attending physician or psychologist without preselection. EbEs were recruited via snowball sampling throughout Germany [35]. Recruitment was terminated once the interviews of the patient and expert groups had reached thematic saturation [35]. All participants received and signed informed consent, and there was no compensation for participation.

### Data collection

A total of 26 problem-centered interviews [36] were conducted by PN (*n* = 30) and MW (*n* = 1) between October 2021 and February 2022 using a semi-structured interview guide. Both had clinical experience, although PN was still a medical student at the time of the interviews. Some participants explicitly valued this as a source of impartiality.

The interview guide was developed based on the authors’ clinical experience, prior research and in consultation with a multidisciplinary Qualitative Research Workshop (QRW) [37] consisting of psychologists, sociologists, physicians and other social scientists to reduce bias and increase validity.

As part of this process, feedback was obtained from a researcher with lived experience. This consultation aimed to critically review the guide and to identify and remove topics that might not be relevant or appropriate from a patient perspective in this exploratory study. The feedback was incorporated into the final version of the guide. While the consultation did not introduce entirely new themes or alter topics, specific adjustments were made to the guide based on the experts’ feedback (e.g. questions were rephrased for clarity). The researcher was involved in a single consultation and did not receive financial compensation.

The guide was tested in a pilot interview, which was also included in the study [37]. Following this, the guide was adapted for the subgroups of EbEs.

The guideline includes their own concept of mental illness, medication, their views on help from treatment providers, involvement of relatives, accommodation, coercive measures, stigmatisation, work, research, and the need for research (the interview guide is available online). In this manuscript, we will report on aspects of the view on research and potential research topics only.

Questions about research were, for patients only, preceded by asking whether the respondent had a general idea of mental health research. EbEs and patients who confirmed this were then asked about their image of research, how studies should be conducted, whether they were informed about current studies, and what their own research would entail if they had their own institute. Finally, participants’ sociodemographic data [36] were collected.

The interviews lasted on average 59 min (9–126 min, median 64 min; EbE: 71 min, patients: 43 min) and were transcribed and anonymised (PN). All interviews with patients were conducted face-to-face, and most interviews with experts were conducted via encrypted video calls.

This manuscript focuses primarily on EbEs, as the results showed that their accounts of research and research needs were the most detailed and conceptually elaborated. Experts conducted significantly longer interviews than patients did (71 vs. 43 min) and produced more substantial findings. This supports the initial rationale for including this group, as EbE are uniquely positioned to reflect on research through the combination of lived experience and experiential or professional engagement with mental health services and research.

Rather than indicating a hierarchy of perspectives, this reflects the specific analytical focus of the present study on how research is perceived, understood and should be. Including both stakeholder groups equally in this analysis would have resulted in a strong imbalance in the depth and scope of the responses. We therefore chose to focus on EbEs in order to provide a more coherent and in-depth analysis of research-related perspectives.

At the same time, findings from patients are drawn upon where they offer contrasting or complementary perspectives, in order to contextualise and enrich the analysis.

### Analysis

The interviews were analysed using MAXQDA analysis software (Versions 2020 and 2022) according to Kuckartz’ qualitative content analysis [34]. The initial analysis categories were derived deductively from the interview guide. These were used to code the entire material, with further categories and subcategories being formed inductively (PN). The category system was then jointly discussed and revised (PN, MW). With the revised category system, all codes were checked again and recoded if necessary.

The interim results were repeatedly discussed in the QRW and presented in a doctoral colloquium to critically scrutinise the authors’ interpretations in the multiprofessional forum and increase the validity of the data [38].

### Patient and public involvement

In this study, the direct involvement of people with lived experience in the design phase was limited to consultation. Although no formal co-production approach was implemented, the interview guide was refined based on critical feedback from one researcher with experience in psychiatry to ensure its relevance to participants. To document this involvement transparently, we applied the GRIPP2 Short Form checklist [39] (completed checklist is available online). The study itself focuses on eliciting and analysing the perspectives of people with lived experience and other stakeholders regarding research needs in psychiatry.

### Participant demographics

There were *n* = 13 EbE, and *n* = 13 patients; 13 participants were female (see Table 1). The EbE group consisted of 12 peer support workers and one researcher with psychiatric experience. Several of the peer support workers had participated in studies or worked in research. Five EbEs had been diagnosed with schizophrenia, three with schizoaffective disorder, three with depression, one with bipolar disorder, and one with borderline personality disorder and substance use disorder. Experts without a diagnosis of schizophrenia or schizoaffective disorder had professional roles (e.g., as peer support workers on acute wards or as researchers) that brought them into contact with these disorders, thereby acquiring relevant experience.

Patients were recruited after being identified by the ward physician or psychologist as having a clinical diagnosis of schizophrenia or schizoaffective disorder (ICD-10: F20, F25). Nine patients reported having been diagnosed with schizophrenia and two reported having been diagnosed with schizoaffective disorder. One participant was unaware of their specific diagnosis, and another did not provide sociodemographic information, including information on their diagnosis.

**Table 1:** Sociodemographic data of the participants

		Total Sample				EbE		Pat	
		*n*				*n*		*n*	
**Total**		26				13		13	
Gender									
Female		13	(50%)			8	(55%)	5	(45%)
Male		13	(50%)			5	(45%)	8	(55%)
**Demographic characteristics**		25¹				13		12²	
Age (in years), mean		48,04				52,8		42,8	
Marital status									
Single		13	(52%)			7	(54%)	6	(50%)
Married/ In a partnership		7	(28%)			4	(31%)	3	(25%)
Divorced		4	(16%)			1	(8%)	3	(25%)
Widowed		1	(4%)			1	(8%)	-	
Children									
Yes		9	(36%)			6	(46%)	3	(25%)
No		16	(64%)			7	(54%)	9	(75%)
Employment Status									
Employed *(not as peer support worker)*		5	(20%)			1³	(8%)	4	(33%)
Peer support worker		11	(44%)			11	(85%)	-	
Disability pension		6	(24%)			-		6	(50%)
In education/training		1	(4%)			-		1	(8%)
Homemaker		1	(4%)			1	(8%)	-	
Unemployed		1	(4%)			-		1	(8%)
Highest level of education⁴									
University degree		4	(16%)			3	(23%)	1	(8%)
Vocational Training		16	(64%)			9	(69%)	7	(58%)
High school diploma (Abitur)		4	(16%)			1	(8%)	3	(25%)
Secondary school certificate (Mittlere Reife)		1	(4%)			-		1	(8%)

Note:

¹ All other variables despite gender: *N* = 25 (one missing value)

² All other variables despite gender: *N* = 12 (one missing value)

³ This EbE is working as a researcher with lived experience

⁴ Except for formation as a peer support worker

### Translation and editing

We used DeepL for the translation of non-English sections and Le Chat and ChatGPT for language refinement. All AI-assisted content was reviewed and approved by the authors.

## Results

The data from all 26 interviews revealed 18 main themes, reflecting the range of topics included in the interview guide. This manuscript focuses on different views on research – one of the 18 main themes. The results focus on EbEs but are enriched by the perspective of patients (cf. Methods/Data collection).

Three categories related to perceptions of research emerged (see Table 2): ‘the image of mental health research’, ‘who should do research’, and ‘manner of research’. In total 12 EbEs and 9 patients spoke about these topics. The categories emerged from the joint analysis of EbEs and patients, with EbEs contributing the majority of the material. Two subcategories, ‘1.4 Historical development of psychiatry and possible influences of research’ and ‘3.2 Structural framework for research’, emerged exclusively from EbE accounts, whereas several others were also predominantly based on EbE accounts (see Table 2).

**Table 2:** Category system

Category	Subcategory	Frequency	
		Participants (Exp/Pat)	Numbers of Segments
1.) The image of mental health research	Totally:	25 (12/9)	69
	1.1 Neurobiological themes	12 (6/6)	23
	1.2 Psychotherapeutic and psychosocial themes	3 (1/2)	4
	1.3 Quality and quantity of research	5 (4/1)	6
	1.4. Historical development of psychiatry and possible influences of research	11 (11/0)	24
	1.5 Own experience with research	9 (7/2)	11
2.) Who should do research?	Totally:	13 (9/4)	27
	2.1 ‘all departments’	6 (5/1)	6
	2.2 Professional expertise	9 (6/3)	11
	2.3 Involvement of people with psychiatric/crisis experience	8 (6/2)	10
3.) Manner of research	Totally:	8 (6/2)	57
	3.1. The topics and the way of carrying out the research	7 (6/1)	24
	3.2. Structural framework for research	3 (3/0)	11
	3.3. Lived experience	5 (4/1)	22
Total		21 (12/9)	153

### The image of mental health research

This category is about participants’ image of mental health research: On the one hand spontaneous associations on research, prompted by the question: ‘What image comes to mind when you think of science in psychiatry or studies in psychiatry?’ and on the other hand on their perspective of the development of clinical psychiatry and the influence of research. This category included comments from the majority of all study participants, which is why quotes by both participant groups are provided below.

#### Participants’ relationship to research

The range of associations on mental health research was wide, with some participants having just vague ideas of research and its conduct and others – especially EbEs – having more specific ideas and experience of research:

Science [means] that all of that needs to be tested, or better… [8 sec pause] I don’t know. (No. 1, Patient (Pat))

Those with a more specific concept of research have often participated in many projects as participants or co-researchers:

I can think of studies that look into diagnoses and disease patterns. I think that was also my first contact, because I volunteered to take part in studies and participated in some. Sometimes it was just an interview, sometimes it was really complex with CT scans and all sorts of things. And there were very different experiences there, too. (No. 23, EbE)

#### Neurobiological topics

In general, associations on basic neurobiological research were not so common. However, thoughts and desires about medication were mentioned by almost half of the participants. They suppose, *‘that perhaps something can be improved with tablets’* (No. 4, Pat) or with the ‘*formulation with chemicals*’ (No. 14, Pat). One participant also states that psychological research is needed but emphasises that ‘*drug development is incredibly important*’ (No 22, Pat).

When asked directly about medication, the majority of participants reported problematic side effects. Five participants explicitly expressed a desire for more research into this issue.

One EbE reflects on the limits of neurobiological research in the context of their own earlier academic career, which they conducted for a short time as a doctoral student in the field of brain research. They are also interested in participatory research and state the limits of neurobiological research.

We can’t really understand [the brain], because it’s always bigger than us - because we are not its creators, we haven’t experienced that ourselves, so… I sometimes find that approach almost a bit arrogant, when people think that by studying biological and neurobiological processes they can find the ultimate truth. (No. 6, EbE)

#### Psychotherapeutic and psychosocial subjects

Interest in neurobiological research is often shown alongside interest in psychotherapeutic or psychosocial issues and the two are not considered to be contradictory:

[…] that maybe you can improve something with tablets, or that you can improve the way you interact with people (No. 3, Pat)

An EbE states:

[…] that one aims for improvements in how to deal with patients, or how to conduct psychotherapy better. That there’s an effort nowadays to make psychiatry more modern. (No. 7, EbE)

Several participants define ‘modern’ psychiatry as a form of psychiatry associated with psychosocial and psychotherapeutic approaches. One participant defines modern psychiatry as a discipline that treats patients with respect and aims to establish a collaborative partnership (see also [33]).

#### Quality and quantity of research

Two participants criticise the quality and quantity of research in psychiatry as being insufficient. One participant describes the discrimination and stigmatisation of people with mental illness, which they also observe within the field of research. They compare psychiatry with other disciplines and state that, ‘*people with mental illness always fall through the cracks. And I also feel*,* or I experience it that way*,* that we often need help that we don*’*t even get’* (No. 15, EbE).

In general, participants regret that progress in mental health research needs a lot of time:

Things have definitely changed, but everything is moving way too slowly. I had to realize that too — after two, three years I noticed, ‘OK’, I was probably a lot more optimistic about how quickly things would change. Right. Had to realize that things are changing, but it takes a very, very long time. (No. 23, EbE)

Furthermore, some participants argue that insufficient funding is allocated to mental health research. One participant articulated a perceived structural contradiction between research aimed at improving patient outcomes and an institutional logic of care provision that relies on the persistence of illness:

There are also huge financial interests here […]. I’m being a bit cynical here. [What would happen if] patients learn very well to take care of themselves, to get their lives under control, to independently get the help they need. Then some institutions would actually lose their clients. They wouldn’t like that at all. Of course, there are significant obstacles and braking mechanisms in the system. (No. 8, EbE)

#### Historical development of psychiatry and possible influences of research

Nevertheless, most participants shared the impression that psychiatry ‘*has become more humane in recent decades*’ (No. 23, EbE). An EbE who trained as a nurse over 40 years ago states: ‘*30 or 40 years ago*,* […] people were simply restrained or sedated*,* a lot has changed and I think this development is extremely important*.’ (No. 10, EbE). Another EbE attributed this change to ‘*empowerment*,* a growing self-help movement*,* people with mental illness standing up much more and*,* and also standing up for their rights*’ (No. 8, EbE).

Participants observe progress in several areas, including medication (lower doses, less sedation), patient autonomy, the (decreased) use of restraints, mental illness stigma, and the relationship between patients and staff. On the other hand, one expert observed that things have not changed enough: For them, there is still too much distance between staff and patients.

I was in psychiatry for the first time back in 1970, so you can definitely say it’s not as bad today as it was then. But a certain attitude, or a certain way of dealing with the concept of illness — like, ‘that has nothing to do with us [= the staff], we just have to treat them’ — that kind of, I think, wrong approach, well, that still exists. (No. 9, EbE)

Finally, two EbEs questioned whether the core issue lies in a lack of research or rather in its insufficient implementation:

But at the end of the day… my problem is that I think when so much research has been done and I know psychiatrists that I think are doing a good job […], but I work in a hospital where I don’t see these research results implemented. Research is only interesting if it doesn’t exist in isolation, but if it’s applied in practice. In that sense, it becomes consequential. (No. 9, EbE)

### Who should do research?

The second category addresses the question of who should conduct mental health research. Participants were asked directly who should be involved in mental health research.

#### ‘all departments’

Participants state that research teams should be ‘*multi-perspective*’ (No. 23, EbE). One EbE discusses that the composition of research groups should depend on the research topic, but also include social sciences:

Philosophy and sociology, in particular, always form important interfaces with psychology and psychiatry, because fundamental questions of, for example, ethics, always arise. Because questions of social interaction, including social values, always arise, or at least should always arise, in the background. (No. 8, EbE)

#### Professional expertise

Regarding researcher training, several respondents emphasize the importance of having ‘*learned how to work scientifically during their formal education*’ (No. 4, EbE). One EbE explained that they are very interested in research but could not pursue it because ‘*you need an academic background for that*’ (No. 9, EbE). Another participant expressed this pointedly: ‘*Nobody can work as a bar manager or kitchen manager who is not a kitchen manager*’ (No. 20, Pat).

However, some EbEs later revised their statements: ‘*It can be anybody*,* it can be a peer support worker; I’ve been on a [research] trial before*’ (No. 4, EbE). Interestingly, one participant who initially emphasized the need for formal training had already conducted research themselves as a peer support worker. This may reflect the persistence of hierarchical structures in psychiatry that can lead individuals to devalue or delegitimize their own research contributions.

In contrast, one EbE emphasized the value of including people without an academic background.

I would also like people without an academic background to be involved, because sometimes they may have a more naive view, which is not so bad, or because they ask a question that is no longer being asked internally. And apart from that, I’m not so radical that I would - if research was open, if research wasn’t so fixed from the outset. (No. 9, EbE)

In addition to those without an academic background, it is also desired that those involved in research have a background in mental health practice too:

They are practitioners saying that something has to change here. Those are often people with a great deal of courage and commitment and they get things moving. And they then get the ball rolling and this in turn can and must be researched. And there is also accompanying research. But the ideas can really only come from people in the field or from people with practical experience, because they know what is needed. That’s just logic. (No. 8, EbE)

Another participant proposes to include legal carers, ‘*because they are very close to the patients*’ (No. 7, EbE).

#### Involvement of people with psychiatric/crisis experience

There is a strong desire for greater involvement of people with psychiatric or crisis experience. One participant states they ‘*would actually see […] it as a great opportunity if patients were trained to participate in research*.’ (No. 5, Pat). Another participant suggests that ‘*relatives are very much interested in getting involved*’ (No. 20, Pat). An EbE wishes: ‘*Persons being researched should have their say. By all means.*’ (No. 9, EbE).

One expert argues that a different approach is needed in dealing with researchers’ own psychiatric experiences, so that it would no longer be necessary to rely on external peer researchers:

It would be extremely helpful if we had a scientific system that allowed researchers who have had their own experience of mental illness to come out. [...] Of course, it’s not at all the case that you have to bring in people with experience of psychiatry from outside and train them painstakingly to bring that experience into the scientific system. If you look at the prevalence of mental illness, it is perfectly logical that as soon as you have a larger group of researchers, you will have people who have experience of illness. And actually everybody knows that, the crazy thing is that nobody dares to admit it. Or almost nobody. Because they’re simply afraid that their careers will be seriously jeopardised. And then, in my opinion, this absurdity sets in: Unfortunately, we don’t have any experience in our system, and now we have to bring in people from outside and train them painstakingly, knowing that they will never be able to really act scientifically on equal terms, because they don’t have the background, the extensive training. And that creates a skewed position that is completely unreal. (No. 8, EbE)

Another EBE emphasizes that relevant lived experience does not necessarily have to involve a diagnosed mental illness, but can also stem from an awareness of having gone through a life crisis:

People who are open about their fragility, their own fragility perhaps. So people, I don’t think they have to be psychiatrically experienced, they don’t have to have had major crisis experiences in their lives, but everyone knows crises, everyone knows breakdowns and they have to take that into account and find a good way of dealing with it. I think I would like to find a good way of dealing with it, in other words not to hide behind the professional role, which sometimes happens in psychiatry. (No. 18, EbE)

### Manner of research

The third category covers topics of research, how research is conducted, the circumstances of research and the inclusion of lived experience in research.

#### Topics and ways of conducting the research

Although participants favour research being conducted in general, many are dissatisfied with the specific topics and methods employed:

Interestingly, research really annoyed me, because I always thought that we have been researched an incredible amount, […] the questions that are asked are wrong, or I find it strange that we always have to be available to say things, and […] it’s not what’s interesting, and it’s not what will help to improve mental health care. (No. 9, EbE)

Participants want people with lived experience to be able to influence the agenda so that the research topic has an impact on people’s daily lives and treatment:

And then I would like people who have experienced this, to name the need for research, so that they are not ignored and the needs of the people are not ignored. (No. 18, EbE)

On the other hand, this participant points out that basic research is also important:

Basic research is also very important and is needed. And every researcher should of course follow their curiosity and their thirst for knowledge, which pulls them in a certain direction. (No. 18, EbE)

There is concern over the pre-definition of research outcomes:

But I’m not criticising research in principle, I just sometimes have the feeling that something specific is supposed to come out of it and… and it doesn’t help to overcome this, this, this crypt [between professionals and patients] as long as it goes on like this. (No. 9, EbE)

The treatment of patients within research settings is criticised in relation to the conduct of studies:

And I think I was particularly annoyed during research. Sometimes I thought, what right do you have to ask me that? I’m not an alien. (No. 9, EbE)

The metaphor of the ‘alien’ conveys the participant’s sense of otherness, highlighting the perceived detachment and lack of sensitivity in their treatment and experience as a study participant. This sense of otherness is also reflected in the earlier metaphor of the ‘crypt’, a symbol of the mysterious, occult and realm of death, which further emphasises the perceived distance and inaccessibility of the research context. Another participant stressed that the interpersonal relationship between the investigator and the participant is also an important aspect of research studies.

One participant expressed frustration with superficial data-gathering methods that create the illusion of inclusion without giving meaningful consideration to the input of participants:

What really annoys me are these pseudo-surveys where they say, well, we’ll add a small text field to the questionnaire so that the clients have the nice feeling that they are also being asked about their experiences, but unfortunately we can’t take this into account in the evaluation because we don’t have the capacity to do so. (No. 8, EbE)

For participants, it is important that research leads to tangible benefits for patients. There is an expectation that studies should be application-oriented and contribute to positive patient outcomes. Additionally, concerns are raised about the allocation of research funding and resources when they do not result in improvements to patient care.

I just think, this research should put something into practice in the end. […] it should be research that ultimately benefits the patient and is application-orientated. That’s how I see it. (No. 26, EbE)

I get that too, because I work at the [hospital], which is a university hospital. And sometimes I get the feeling that ‘you guys are really crazy about what you spend money on’. In fact, sometimes I’m completely blown away and think that what you’re doing, where you’re spending an incredible amount of money and employing an incredible amount of people, isn’t changing or improving the situation of people on psychiatric wards. '' […] it doesn’t really make anything better (No. 9, EbE)

#### Structural framework for research

Participant also have doubts about the structural framework of research.

One expert reports that they no longer trust the neutrality or truthfulness of research:

Instead, I took part in other projects that were researched afterwards. And then I read the reports. And since then I’ve been much less impressed by research anyway, because I thought, hey, you’re claiming things – I just know, I was there, that it’s not true. […] It’s not just a question of interpretation, but things are claimed, for example, that we met regularly as a study group. And that’s exactly what we didn’t do, and that’s what we needed. I think I used to be so impressed. And that was so untouchable. I think research is goal-orientated, it’s not neutral, it’s not open. Something specific is supposed to come out of it and it is, so to speak, allowed – I’m thinking of another project right now that I think went wrong. I don’t think that’s such a bad thing because you learn a lot from it. And you shouldn’t sugarcoat it. (No. 9, EbE)

The sustainability of research and research-related interventions is also criticised due to a lack of funding:

then it’s a project for which there was funding for three years and then it wasn’t funded any further in that, in that and then it no longer existed and … that’s also something political again… because even if it still existed now, it would still be a project and it would actually fill a gap in care. (No. 23, EbE)

A researcher with lived experience complains that the high-impact publishing system hinders research on topics that are relevant to practice but don’t receive recognition in the scientific system:

It has long been proven, for example, that the relationship is the most important factor of all. Unfortunately it still plays a relatively minor role in research, probably because it is also difficult to research, something as complex and ‘soft’ as a relationship cannot simply be tested with an RCT. And then we run into the problems of research logic, where people prefer to investigate what they can use to achieve high-ranking publications and research fame. This is an inherent logic of research, but it means that the topics that are important at grassroots level are often left out. And I actually find that very regrettable. (No. 8, EbE)

Furthermore, this researcher states that this not only affects the choice of topics, but also the quality of the research. According to them, a lot of effort is put into ‘*data from the RCT*’ (No. 8, EbE), while qualitative studies, which are sometimes required for research proposals, are carried out poorly or inadequately.

According to this EbE, the issue of satisfaction also affects participatory research:

Participatory research — all the many conversations you have to have. If you take seriously what I just said, about first getting a comprehensive picture of practice, meaning talking to everyone involved, that’s a lot of work. And what do you have to show for it? At first, nothing at all. You don’t have a publication yet, let alone a high-ranking one. If you spend your time searching databases, you get much more out of it — at least from a career perspective. And if you then do a quick study with a few surveys and publish it in a top journal, wonderful. But talking to people — and I’m caricaturing a bit here and being a little cynical, but I don’t think I’m wrong in principle — talking to people and being active in that way is not something the research system rewards very much. (No. 8, EbE)

#### Lived experience

EbEs, in particular, stress the importance of including persons with lived experience in mental health research. One participant queries how to deal with lived experience:

What value do we attach to experiential knowledge? And, how much of a mouthpiece do we give it? How much value do we give it in a scientific article, in a journal? […] I think that’s an exciting question anyway: how much value is given to objectivity, to subjectivity, so I don’t think there is any such thing as objectivity anyway. (No. 23, EbE)

In terms of incorporating lived experience, one researcher with psychiatric experience distinguishes between basic research and care research emphasizing the role of empirical knowledge in ensuring validity:

I think basic research has different foundations and prerequisites altogether. But when it comes to health services research, ecological validity, in my view, can only be achieved if you include experiential knowledge. In an appropriate way and explicitly label it as experiential knowledge. Because I believe only then can a scientific approach really continue to be ensured. (No. 8, EbE)

Participants describe that the involvement of peers changes the process of research and that it is necessary to redefine ways of doing research together. One expert emphasises that it is also important to take a differentiated view of the competence and knowledge of peers:

Like there’s always the danger of beatification of those affected, which is nonsense. We’re not better people just because we have experience of psychiatry, but we really do have a different perspective, and I don’t think mental health research is possible without including this view. (No. 9, EbE)

## Discussion

This study explores how experts by experience (EbE) perceive mental health research in the context of schizophrenia, enriched by the views of patients. For this purpose the study takes a broad perspective on research, including agendas, methods, and forms of patient involvement, as evidence in this area remains limited [28, 29]. Our findings contribute to improving the agenda setting, research methods, and the involvement of people with lived experience, in order to make research more relevant and facilitate translation into practice [2, 5, 9, 18, 19]. This aligns with Ludwik Fleck’s [6] observation from as early as 1935 that research is never purely objective but shaped by ‘thought collectives’ influenced by social and political incentives – but not necessarily aligned with the needs of those affected. This highlights the importance of including the voices of EbEs and patients.

### *Image of psychiatric research*

In contrast to prevalent research agendas [4, 9], participants rejected the primacy of neurobiological approaches in psychiatry. While they acknowledged the importance of medication, they equally valued psychotherapeutic and psychosocial research – a preference consistent with previous studies [2, 3, 10, 27]. The participants held the view that progress in mental health remains slow and limited, partly because of limited implementation. Given the dominance of neurobiological approaches, this underlines the need for greater emphasis on psychosocial research – and its translation into practice. Beyond the question of what psychiatric research should focus on, participants also reflected on who should be involved in conducting it.

### *Who should do research?*

While most participants advocated for multiperspective teams, a subgroup expressed scepticism about including non-academics – a tension that highlights the need for training and support to enable meaningful EbE involvement. Consistent with previous findings [29], there is a strong desire to involve peer researchers and lived experience in research, and participants prefer professional researchers who are committed to patient needs and research across boundaries [5].

The involvement of lived experience and service users can be empowering: studies report that service users take their role very seriously, are willing to make a significant time commitment, and feel motivated by their wish to help medicine and other people with illnesses [19]. They report that their opinions and experiences are mostly taken seriously and that they are satisfied with their involvement [19, 40]. Involvement in research has been described as a means of empowerment: people with lived experience no longer feel like ‘research subjects’ but rather as researchers themselves [40].

This finding is all the more encouraging given that participants in our study complained about poor involvement in studies or about results not being taken seriously. Achieving comprehensive involvement of service users in research is challenging [19], whereas merely gaining tokenistic participation in a few formal meetings is not sufficient [41]. It is crucial that researchers take involvement seriously by assigning substantive roles and responsibilities, ensuring adequate time for collaboration, and minimizing professional hierarchies during interactions [19, 40].

In a study on bipolar disorder, clinician-scientists successfully collaborated with service users to develop research questions, with some user priorities proving novel compared with clinician-generated ones [31]. This demonstrates that EbE involvement can enrich research agendas – a potential that also exists for schizophrenia research.

### *Manner of research*

With regard to research topics, there is a strong desire for user-set priorities, which is also in line with the recommendations of Chalmers et al. [9] for reducing useless research as well as the growing number of studies exploring research priorites, including the opinions of service users [11]. However, many of these studies still prioritise the views of ‘clinicians, academics and government rather than [of] people with lived-experience” or their relatives [20]. EbEs criticised the research questions as being incorrect or ‘strange’, which corresponds with earlier studies, in which service users criticised research questions as being too narrow and argued that they should be broader and more holistic [19]. There is a concern that research is not always neutral, but is sometimes predetermined or biased. The lack of sustainability in research and research-related interventions due to inadequate funding was also criticised by participants.

One key concern for participants was that research should yield direct and tangible benefits for psychiatric patients. However, there is criticism that the peer review system and the structural framework favouring RCTs do not always lead to the most useful research. This corresponds with concerns among peer researchers in the literature that researchers focus more on publishing than on the project itself [40]. Our participants extended this critique to financial dependencies as a key barrier to independent research. While Wannheden [40] highlights academic career incentives as a threat to research integrity, our EbE emphasised that funding structures compromise the neutrality and relevance of psychiatric research.

As in earlier studies [3, 40], participants articulated a strong desire for the inclusion of lived experience and the need to explore how to deal with this experience. According to Wannheden [40], the question should be ‘how to create equal partnerships’ in research, as concepts like *co-creation* or *partnership* are said to be more participatory than just ‘involving, engaging or empowering patients and public stakeholders’ [40]. Regarding the inclusion of lived experience, five important factors are recommended by Dray et al.: ‘lived experience as a critical ingredient; moving from research participation to active involvement; […] flexibility, choice and agency [in the participation]; sharing experiences, stories and backgrounds; and power, value and voice’ [29] of the researchers with experience.

Additionally, some respondents proposed disclosing the personal experience of professionals in research teams, so that people without scientific experience (but with lived experience) would not need to be trained in research. As Banfield et al. reported, ‘academic researchers who also have lived experience have the potential to bridge the gap between the consumer sector and traditional academic researchers’ [28]. However, it is important to recognise that lived experience varies greatly. For example, the perspectives of academic researchers may differ substantially from those of individuals who have not had the opportunity to pursue an academic career. Following this, it would be beneficial to empower people without an academic background to take part in research as well [42]. As Banfield et al. argue everybody has different ‘identities and intersecting expertise’ [42], making it essential to consider a wide range of perspectives and expertise [43] including both academic and non-academic EbEs.

Regarding the development of psychiatry, some participants stated that there is change, but it is slow and insufficient. Some participants highlighted the poor quality and quantity of mental health research in general. A lack of or slow progress in psychiatry has often been mentioned by researchers as well [1, 5, 17–19], but to our knowledge this perception has not yet been documented from the perspective of people diagnosed with schizophrenia themselves. In contrast to our findings, Morse et al. reported that mental health service users thought that research plays a significant role in advancing psychiatry [31].

One participant described feeling like an ‘alien’ (No. 9, EbE) in research settings, which aligns with Morse et al.’s [31] findings on stigma related to participating in psychiatric studies, but extends this to a structural critique of power imbalances in psychiatric studies, by describing a ‘crypt’ (No. 9, EbE) between them and professionals, and by expressing a desire for better treatment studies.

Finally, one participant wondered whether the main problem was an implementation gap. This concern has already been raised [2, 8]. As Maassen et al. point out, the lack of implementation could also be viewed as a research gap identified by service users [44].

## Limitations

Regarding sampling, EbEs were recruited from all over Germany. In contrast, patients were recruited from one hospital in southern Germany, which could have reduce the diversity of patients’ experiences. While EbEs had diverse work and treatment experiences in various mental health settings, it should be critically noted, that the recruitment took place via four contacts using the snowball method, which entails the risk of a certain degree of sample homogeneity.

Patients were included solely on the basis of their diagnosis and were recruited consecutively, without preselection of socio-demographic factors or severity of illness. Nevertheless, inpatient recruitment typically results in a sample of more severely affected individuals, which helps to include the voices of those who are usually not heard.

With respect to the aim of achieving comprehensive participant involvement, it can be stated that one expert was involved in the consultation process to develop the interview guides. Greater involvement of stakeholders in the planning, implementation and evaluation of the study would have been desirable to better represent and reinforce the perspective of people with lived experience. At the same time, this might have led to a certain circularity, as the aim of this study was to listen to their narratives as participants with an open mind regarding themes and opinions.

While patients offered valuable additional insights, most of the in-depth reflections on the research question and comments on the relatively abstract nature of the topic of this paper were provided by EbEs. These individuals have extensive treatment experience and are actively engaged in reflecting contexts of mental health care. Many have also participated in clinical trials or worked as peer researchers. However, it is questionable whether this focus is appropriate, or whether the aim should be to incorporate the perspectives of patients more. It is debatable whether this focus occurred because of the open, semi-structured interview format which may be less effective for this stakeholder group for gathering information on such an abstract topic, or because non-scientific personnel struggle to answer such questions.

## Conclusion

In this study, we aimed to explore how peer support workers and researchers with lived experience, amplified by the perspective of people with schizophrenia or schizoaffective disorder, perceive current mental health research and its future research needs in the context of schizophrenia. Our findings confirm and extend previous research by highlighting the central role that lived experience plays in participants’ views on mental health research. Three key areas for improvement were identified: (1) greater involvement in defining research priorities, (2) comprehensive inclusion of people with lived experience in research teams, and (3) openness and disclosure of researchers’ own psychiatric experiences.

While participants acknowledged progress in psychiatry attributable to research, they perceived this progress as slow and insufficiently implemented in practice. Additionally, concerns have been raised about how patients are treated in studies and whether their contributions—particularly in qualitative studies—are taken seriously.

To address these gaps, our study recommends strengthening the involvement of people with lived experience across all stages of the research process, particularly in countries like Germany, where participatory approaches are still emerging. Furthermore, the manner in which participants are treated in studies should be improved, and their contributions should be taken seriously.

It is necessary to continue and reinforce the involvement of people with lived experience in research processes to reduce research waste and close the implementation gap.

## Supplementary Information

Below is the link to the electronic supplementary material.

Supplementary Material 1: GRIPP2 short form.docx: The GRIPP2 reporting checklist in the short form.

Supplementary Material 2: Interview guide.docx: Interview guide: These are the interview guides used to interview the experts by experience and patients.

## Data Availability

The qualitative interview transcripts generated and analysed during the current study are not publicly available due to their sensitive nature and the risk of participant identification. Anonymised quotes relevant to the study are included in the article, also the category system. Further data may be made available from the corresponding author on reasonable request, subject to ethical approval and data protection regulations.

## Abbreviations

EbE: Experts by psychiatric experience (peer support workers or researchers with psychiatric experience)
MW: Moritz Wigand
Pat: Patient
PN: Paul Nickel
PPI: Patient and Public Involvement
RCT: Randomised Controlled Trial
QRW: Qualitative Research Workshop
