# Supplementary material for: Views on mental health research in the context of schizophrenia: perspectives of peer support workers and researchers with lived experience – a qualitative study
Source: Res Involv Engagem. 2026 Sep 23;12:151. doi: 10.1186/s40900-026-00975-5 (PMC13599242; doi:10.1186/s40900-026-00975-5)
Supplement: Supplementary file 1 [file 40900_2026_975_MOESM1_ESM.docx]

**GRIPP2 short form**

| **Section and**  **topic** | **Item** | **Content** | **Reported on page No** | |
| --- | --- | --- | --- | --- |
| 1: Aim | Report the aim of PPI in the study | To improve the interview guide and ensure that patient-relevant questions were included. | | 8 |
| 2: Methods | Provide a clear description of the methods used for PPI in the study | A researcher with experience in psychiatry provided feedback on the guide. For this, the drafts of the patient and relative guides were emailed to them and they returned them with suggestions for improvement. | | 8 |
| 3: Study results | Outcomes—Report the results of PPI in the study, including both positive and negative outcomes | Specific adjustments to the interview guide were made based on the expert’s feedback (e.g., rephrasing questions for clarity). | | 8 |
| 4: Discussion and conclusions | Outcomes—Comment on the extent to which PPI influenced the study overall. Describe positive and negative effects | The influence of PPI was limited to the development of the guide. Here the consultation did not identify entirely new themes, thus its impact on the final guide was limited. However, the feedback improved the guide and confirmed the relevance of existing questions. | | 8 |
| 5: Reflections/critical perspective | Comment critically on the study, reflecting on the things that went well and those that did not, so others can learn from this experience | We involved one expert by experience in consulting on the interview guide development. Greater stakeholder involvement in the planning, implementation, and evaluation could have enhanced the representation of lived experience perspectives. However, broader involvement might have introduced circularity, as the study aimed to capture participants’ narratives without preconceptions. | | 28 |

1. *PPI* patient and public involvement
