# Supplementary material for: Views on mental health research in the context of schizophrenia: perspectives of peer support workers and researchers with lived experience – a qualitative study
Source: Res Involv Engagem. 2026 Sep 23;12:151. doi: 10.1186/s40900-026-00975-5 (PMC13599242; doi:10.1186/s40900-026-00975-5)
Supplement: Supplementary file 2 [file 40900_2026_975_MOESM2_ESM.docx]

**Interview Guide for Expert by Experience Interviews: Research Needs in Psychiatry from the Perspective of People with Schizophrenia^[1]^**

| Thank you very much for taking the time to participate in this interview.  Together with the University of Ulm, we are conducting a study on the topic *“Research Needs in Psychiatry.”*  The aim of the study is to include your personal experiences as an expert with lived experience with your own mental health history in order to highlight where you see a need for change or have wishes regarding psychiatry and research.  [Information on voluntariness and data protection]  The interview will take approximately one hour. If at any point the interview becomes too exhausting for you, or if you wish to stop for any other reason, we can pause or end the interview at any time and, if you wish, continue it later.  With your permission, I would like to audio-record the interview. Excerpts from the interview may be used in anonymised form, meaning that it will not be possible to identify you, and may be included in scientific publications. Is that acceptable to you?  The audio recorder is now on. Could you please confirm once again that you agree to the recording of the interview, that you have been informed about data protection, and that your participation and all information you provide are voluntary?  Thank you. | | |
| --- | --- | --- |
| **Content Focus** | **Main Question** | **Follow-up Question** |
| **Start of the interview**  Personal background of the expert | Firstl, I would like to ask you: How did you become involved in psychiatric research or as a peer? | What prompted you to do this? How did you find your way into research/into working as a peer? |
|  | What professional background do you bring to your research/your work as a peer? Does this influence your perspective on and approach to psychiatric research/your work? |  |
|  | What do you think - how do your own lived experiences influence your research or work? |  |
| **Research needs**  Understanding of research | I’d now like to talk more generally about psychiatric research: What kind of picture do you have in mind when you think about research or studies in psychiatry? |  |
|  | And who do you think should be involved in conducting scientific studies in psychiatry? Who should act as researchers? | And who should determine the topics? |
|  | Where do you currently see research needs in psychiatry? |  |
|  | Let’s imagine: You are leading a team of scientists and could decide which projects should be worked on in the coming years – what would your ideas be? How would you want these topics to be researched? By whom should these studies be carried out? |  |
| **Need for improvement/research** | Is there anything that, from your perspective, urgently needs to be researched or improved? |  |
| Specific areas | I will now mention several areas of psychiatry. I would be interested in your thoughts on what could be improved or researched in these areas.  Please let me know if there is any area you do not wish to talk about or feel unable to comment on. |  |
| Your own approach to dealing with the disease | How do you view your own experiences of psychosis and your needs as a person affected by the illness? | Do you feel these experiences and needs are adequately taken into account in psychiatry? If not, what would need to change? |
| Understanding of illness | Do you understand the experience of psychosis as an illness?  What does the term *“schizophrenia”* mean to you? | Is there *“healing”* in psychiatry? If so, what do you understand by it?  Do you consider current psychiatric services regarding schizophrenia/psychosis to be appropriate? |
| Medication | What are your thoughts on medication in psychiatry? |  |
| Services, help, and treatment by doctors, therapists, and nursing staff | How do you experience the services, help, and treatment provided by doctors and therapists here in the ward? How do you experience the nursing staff on the ward?  What do you think about outpatient support or help from doctors or therapists? | How is access to these services? Is support needed in finding the right help – for example, following inpatient treatment? |
| Involvement of relatives | What do you think about the involvement of relatives? | Should relatives be involved more, less, or differently? |
| Sexuality^[2]^ | What are your thoughts on sexuality in the context of your illness? | Do you feel that your sexuality or sexual needs are adequately taken into account? |
| Hospitalisation, coercive measures | What is your view on the compulsory admission of patients to psychiatric hospitals in acute situations? What is your opinion on coercive measures? |  |
| Stigmatisation | How do you observe the reactions of the environment and society to your illness? | Do you perceive exclusion or prejudice? Do you experience stigmatisation? Could anything be done to change this? |
| Integration into the labour market | Do you experience a connection between your illness and integration into the labour market? | How could the situation be improved? What would need to change? |
| Support in everyday life | What are your thoughts on support in everyday life? | For example, in relation to housing, structuring daily routines, or dealing with authorities? |
| **Follow-up questions on research** | Do you have the impression that scientific research has brought about changes in support services or treatment options in recent years? |  |
|  | *(only for researchers)* What are you currently researching? How and why do you consider your current work relevant? |  |
|  | *(only for researchers)*  Looking back on your work as a researcher – how would you evaluate the research structures in psychiatry? | Should anything be changed? |
| **Conclusion** | We are now coming to the end of the interview.  Is there anything we have not talked about that you would like to add? | |

**Interview Guide for Patient Interviews: Research Needs in Psychiatry from the Perspective of People with Schizophrenia**

| Thank you very much for taking the time to participate in this interview.  Together with the University of Ulm, we are conducting a study on the topic *“Research Needs in Psychiatry.”*  The aim of the study is to include your experiences with your own illness in order to highlight where you see a need for change or have wishes regarding psychiatry or science.  For this purpose, we are interviewing psychiatric patients in Günzburg.  [Information on voluntariness and data protection]  The interview will take approximately one hour. If at any point the interview becomes too exhausting for you, or if you wish to stop for any other reason, we can pause or end the interview at any time and, if you wish, continue it later.  With your permission, I would like to audio-record the interview. Excerpts from the interview may be used in anonymised form, meaning that it will not be possible to identify you, and may be included in scientific publications. Is that acceptable to you?  The audio recorder is now on. Could you please confirm once again that you agree to the recording of the interview, that you have been informed about data protection, and that your participation and all information you provide are voluntary?  Thank you. | | |
| --- | --- | --- |
| **Content Focus** | **Main Question** | **Follow-up Question** |
| **Current treatment situation and current challenges**  Introduction to the conversation | To begin, could you tell me how you came to be treated in this clinic or in the psychiatric outpatient department? |  |
| Encouraging a differentiated view of one’s own treatment | What experiences are you currently having with your treatment and the support offered here? |  |
| Alternatives to current treatment? | Can you imagine an alternative form of support or help for yourself at the moment? What might that look like? | (If asked: This does not have to correspond to an existing or realistic example.) |
| **Personal illness history**  Reflection on one’s own illness history to identify situations where improved help, support, or treatment options would have been necessary | I would also like to hear about the course of your illness before your current stay. Could you give me a brief overview of your history? | What accompanying effects did your illness have? For example, changes in your social contacts or professional life. |
|  | Could you tell me about the help and support services as well as the treatments you have received thus far? | *If the answer is brief or insufficient:* Please think of all aspects of help, support, or treatment – information, facilities, state support, psychotherapy, social work, family support, hospital stays, medication, etc. |
| **Need for improvement/research** | Is there anything that, from your perspective, urgently needs to be researched or improved regarding support services or the treatment of people with schizophrenia? |  |
| Specific areas | I will now mention several areas of psychiatry. I would be interested in your thoughts on what could be improved or researched in these areas.  Please let me know if there is any area you do not wish to talk about or feel unable to comment on. |  |
| Your own approach to dealing with the disease | How do you view your own experiences of psychosis and your needs as a person affected by the illness? | Do you feel these experiences and needs are adequately taken into account in psychiatry? If not, what would need to change? |
| Understanding of illness | Do you understand the experience of psychosis as an illness?  What does the term *“schizophrenia”* mean to you? | Is there *“healing”* in psychiatry? If so, what do you understand by it?  Do you consider current psychiatric services regarding schizophrenia/psychosis to be appropriate? |
| Medication | What are your thoughts on medication in psychiatry? |  |
| Services, help, and treatment by doctors, therapists, and nursing staff | How do you experience the services, help, and treatment provided by doctors and therapists here in the ward? How do you experience the nursing staff on the ward?  What do you think about outpatient support or help from doctors or therapists? | How is access to these services? Is support needed in finding the right help – for example, following inpatient treatment? |
| Involvement of relatives | What do you think about the involvement of relatives? | Should relatives be involved more, less, or differently? |
| Sexuality^[3]^ | What are your thoughts on sexuality in the context of your illness? | Do you feel that your sexuality or sexual needs are adequately taken into account? |
| Hospitalisation, coercive measures | What is your view on the compulsory admission of patients to psychiatric hospitals in acute situations? What is your opinion on coercive measures? |  |
| Stigmatisation | How do you observe the reactions of the environment and society to your illness? | Do you perceive exclusion or prejudice? Do you experience stigmatisation? Could anything be done to change this? |
| Integration into the labour market | Do you experience a connection between your illness and integration into the labour market? | How could the situation be improved? What would need to change? |
| Support in everyday life | What are your thoughts on support in everyday life? | For example, in relation to housing, structuring daily routines, or dealing with authorities? |
| **Research in general** | Thank you very much for your valuable feedback thus far.  At this point, I would like to know whether you can imagine what science or research in psychiatry might look like? | *(If yes → follow gray-highlighted questions, otherwise proceed directly to the conclusion of the interview)* |
| **Research needs**  Understanding of research | What image comes to mind when you think of science in psychiatry or studies in psychiatry? |  |
|  | And who do you think should be involved in conducting scientific studies in psychiatry? | And who should determine the topics? |
|  | Where do you currently see research needs in psychiatry? |  |
|  | Let’s imagine: You are leading a team of scientists and could decide which projects should be worked on in the coming years – what would your ideas be? How would you want these topics to be researched? By whom should these studies be carried out? |  |
| **Concretisation of research questions** | Are you informed about current or past scientific findings on schizophrenia? | Do you have the impression that the help, support services, or treatments you have received have changed in recent years as a result of scientific research? |
| **Conclusion** | We are now coming to the end of the interview. Is there anything we have not talked about that you would like to add? | |

1. English translation of the interview guides previously published in German as supplementary material to Nickel P, Stiawa M, Wigand ME. Eine Psychiatrie auf Augenhöhe – Beziehungsgestaltung von Fachpersonen aus Sicht von Menschen mit der Diagnose Schizophrenie, psychiatrieerfahrenen Expert:innen und Angehörigen. Psychiatr Prax. 2025;52:214–220. <https://doi.org/10.1055/a-2535-2121>. Translated and reproduced with permission from Georg Thieme Verlag KG. © Georg Thieme Verlag KG.
2. *Content Focus introduced after presentation of first interim results in a Qualitative Research Workshop on 5.11.21*
3. *Content Focus introduced after presentation of first interim results in a Qualitative Research Workshop on 5.11.21*
